# Será a Ablação por Cateter para Fibrilação Atrial em Pacientes com Insuficiência Cardíaca e Fração de Ejeção Reduzida uma Boa Opção Terapêutica?

**DOI:** 10.36660/abc.20230820

**Published:** 2024-02-16

**Authors:** Sergio Menezes Couceiro, Fernando Mendes Sant'Anna

**Affiliations:** 1 Universidade Federal Fluminense Cabo Frio RJ Brasil Universidade Federal Fluminense – Cardiologia, Cabo Frio, RJ – Brasil; 2 Hospital Santa Izabel Cabo Frio RJ Brasil Hospital Santa Izabel – Cardiologia, Cabo Frio, RJ – Brasil; 3 Universidade Federal do Rio de Janeiro Macaé RJ Brasil Universidade Federal do Rio de Janeiro, Campus Macaé, Macaé, RJ – Brasil

**Keywords:** Fibrilação Atrial, Insuficiência Cardíaca, Ablação por Cateter, Metanálise

O artigo foi bem escrito, interessante, tem relevância e validade e teve como objetivo comparar resultados obtidos através de metanálises de ensaios randomizados controlados sobre ablação por cateter em pacientes com fração de ejeção do ventrículo esquerdo inferior a 45%.^1,2^

Os métodos utilizados foram interessantes e procuraram responder à questão principal. Eles buscaram na literatura estudos que comparassem ablação por cateter (AC) com terapia médica em pacientes com fibrilação atrial (FA) com (fração de ejeção do ventrículo esquerdo) FEVE ≤45%. Foi realizada uma metanálise de 7 ensaios clínicos, incluindo 1.163 pacientes com FA e insuficiência cardíaca (IC). Todos os testes foram bilaterais e apenas um valor p <0,05 foi considerado estatisticamente significativo.

O autor utilizou a metodologia PRISMA,^3^ e os dados são incorporados ao artigo de forma comparativa e abordam as 7 metanálises selecionadas.

Foram incluídos apenas ensaios clínicos randomizados prospectivos e os critérios de inclusão foram estudos clínicos que incluíssem pacientes com FA com FEVE ≤45%. O grupo intervenção utilizou ablação por cateter para controle do ritmo (o principal procedimento foi o isolamento das veias). O grupo controle utilizou terapia médica apenas para tratamento, e os estudos relataram pelo menos um desfecho cardiovascular.

Os desfechos clínicos desta metanálise foram mortalidade por todas as causas, hospitalização por IC, recorrência de FA, melhora na FEVE e alterações na distância percorrida no teste de caminhada de 6 minutos (TC6M) e nos escores do Minnesota Living with Heart Failure Questionnaire (MLHFQ).

Isso chama nossa atenção e levanta a questão se a terapia não é uniforme entre os estudos, e nem todos levam em consideração a presença de BRE ou tamanho do átrio esquerdo, o tempo de início e o tipo de FA.

Observamos a necessidade de discriminar a causa da IC isquêmica ou não isquêmica e aplicar uma correção nos resultados.

A comparação dos ensaios CASTLE-AF^4^ e RAFT-HF^5^ revela heterogeneidade que foi corrigida, e a não heterogeneidade foi observada somente após exclusão do ensaio ARC-HF em relação à recorrência de FA.

Somente após ajuste e correção com exclusão do AMICA TRIAL^6^ é que conseguiram obter aumento no tempo de caminhada do teste de caminhada de 6 minutos.

Os autores concluíram que, em comparação com a terapia médica, a AC para FA em pacientes com IC foi associada a um menor risco de mortalidade por todas as causas, hospitalização por IC e recorrência de FA, além de melhorias mais significativas na FEVE e na qualidade de vida, achado que não foi corroborado por estudos mais robustos e com desenho específico.^7-11^

A subanálise revelou que pacientes com disfunção ventricular esquerda mais leve poderiam se beneficiar mais da ablação de FA do que pacientes com doenças mais graves, mas com baixo poder estatístico.
