# Supplementary material for: Será a Ablação por Cateter para Fibrilação Atrial em Pacientes com Insuficiência Cardíaca e Fração de Ejeção Reduzida uma Boa Opção Terapêutica?
Source: Arq Bras Cardiol. 2024 Feb 16;121(1):e20230820. [Article in Portuguese] doi: 10.36660/abc.20230820 (PMC11081178; doi:10.36660/abc.20230820)
Supplement: Translation

# Translation: Is Catheter Ablation for Atrial Fibrillation in Patients with Heart Failure and Reduced Ejection Fraction a Good Therapeutic Option?

**Authors:** Sergio Menezes Couceiro, Fernando Mendes Sant'Anna

**Affiliations:** 1 Cabo Frio RJ Brazil Universidade Federal Fluminense – Cardiologia, Cabo Frio, RJ – Brazil; 2 Cabo Frio RJ Brazil Hospital Santa Izabel – Cardiologia, Cabo Frio, RJ – Brazil; 3 Macaé RJ Brazil Universidade Federal do Rio de Janeiro, Campus Macaé, Macaé, RJ – Brazil

**Keywords:** Atrial Fibrillation, Heart Failure, Catheter Ablation, Meta-Analysis

The article was well written, interesting, has relevance and validity and aimed to compare results obtained through meta-analyses of randomized controlled trials on catheter ablation in patients with left ventricular ejection fraction below 45%.^1,2^

The methods used were interesting and sought to answer the main question. They searched the literature for studies that compared catheter ablation (CA) with medical therapy in patients with atrial fibrillation (AF) with left ventricular ejection fraction (LVEF) ≤45%. A meta-analysis of 7 clinical trials was performed, including 1,163 patients with AF and heart failure (HF). All tests were two-sided, and only a p value <0.05 was considered statistically significant.

The author used the PRISMA^3^ methodology, and the data is incorporated into the article comparatively and addresses the 7 selected meta-analyses.

Only prospective randomized clinical trials were included, and the inclusion criteria were clinical studies that included AF patients with LVEF ≤45%. The intervention group used catheter ablation for rhythm control (the main procedure was vein isolation). The control group used medical therapy only for treatment, and studies reported at least one cardiovascular outcome.

The clinical outcomes of this meta-analysis were all-cause mortality, HF hospitalization, AF recurrence, improvement in LVEF, and changes in 6-minute walk distance (6MWT) and Minnesota Living with Heart Failure Questionnaire (MLHFQ) scores.

It catches our attention and raises the question as to whether therapy is not uniform across studies, and not all of them take into account the presence of LBBB or left atrium size, time since onset, and type of AF.

We observed the need to discriminate the cause of ischemic or non-ischemic HF and apply a correction to the results.

Comparing the CASTLE-AF^4^ and RAFT-HF^5^ trials brings out heterogenicity that was corrected, and non-heterogenicity was observed only after excluding the ARC-HF trial in relation to AF recurrence.

Only after adjusting and correcting with the exclusion of the AMICA TRIAL^6^ were they able to obtain an increase in the walking time of the 6-minute walk test.

The authors concluded that compared with medical therapy, AC for AF in patients with HF was associated with a lower risk of all-cause mortality, hospitalization for HF, and AF recurrence and more significant improvements in LVEF and quality of life, a finding that was not corroborated by more robust studies with a specific design.^7-11^

The subanalysis revealed that patients with milder left ventricular failure dysfunction could benefit more from AF ablation than patients with more severe diseases but with low statistical power.
